# A 50-50% mixture of nitrous oxide-oxygen in transrectal ultrasound-guided prostate biopsy: A randomized and prospective clinical trial

**DOI:** 10.1371/journal.pone.0195574

**Published:** 2018-04-27

**Authors:** Gabriel da Silva Cazarim, Nubia Verçosa, Leonel Carneiro, Rachel Pastor, Elizabeth Fernandes Vaz da Silva, Louis Barrucand, Ismar Lima Cavalcanti

**Affiliations:** 1 Department of Anesthesiology, Federal Fluminense University, University hospital Antonio Pedro, Niterói, Rio de janeiro, Brazil; 2 Department of Anesthesiology, Federal University of Rio de Janeiro, University hospital Clementino Fraga Fihlo, Rio de Janeiro, Rio de Janeiro, Brazil; 3 Department of Statistic, Federal University of Rio de Janeiro, University hospital Clementino Fraga Fihlo, Rio de Janeiro, Rio de Janeiro, Brazil; Eberhard Karls University, GERMANY

## Abstract

**Introduction:**

Transrectal ultrasound-guided biopsy (TUSPB) is the standard method of diagnosis for prostate cancer, and although it is well tolerated by some patients, it presents a discomfort rate of 65 to 90%, which may be associated with pain. For convenience, it is agreed that a method of analgesia and sedation is necessary. For this purpose, this study aimed to evaluate the impact of inhalation of a 50–50% N_2_O-O_2_ gas mixture on pain intensity in these patients.

**Material and methods:**

Randomized, double-blinded clinical trial, conducted at Antônio Pedro University Hospital (Hospital Universitário Antônio Pedro), Niterói, RJ, Brazil, containing two groups of 42 patients: a control (C) group, which received 100% oxygen inhalation, and a nitrous oxide (NO) group, which received inhalation of the 50–50% N_2_O-O_2_ mixture, self-administered during TUSPB. The pain intensity and degree of satisfaction were evaluated through a visual analogue scale (VAS), as was the frequency of adverse events.

**Results:**

Eighty-four patients were included in the study, with 42 in each group. The mean pain intensity was lower in the NO group than in the C group [2.52 (0–10) vs 5.95 (0–10), p < 0.001], and the degree of satisfaction was higher in the NO group than in the C group (8.14 vs. 4.69, p < 0.001). The adverse effects were somnolence, dizziness, nausea, vomiting, discomfort and euphoria without differences between the groups.

**Conclusion:**

The 50–50% N_2_O-O_2_ mixture was effective in reducing pain intensity and increasing the degree of satisfaction in TUSPB, with tolerable side effects.

## Introduction

Transrectal ultrasound-guided prostate biopsy (TUSPB) is the standard method used for early diagnosis of cancer when associated with prostate-specific antigen (PSA) plasma levels[1]. Although well tolerated by many patients, between 65 and 90% of men undergoing TUSPB complain of discomfort[2] associated with pain. Several methods of analgesia and/or sedation have been proposed, including periprostatic[2,3,4,] or intraprostatic[5] nerve block, topical anesthesia with lidocaine[6] or EMLA[7] at the puncture site, and general anesthesia with propofol and remifentanil[8].

The inhalation of 50–50% of nitrous oxide (N_2_O)-oxygen (O_2_) by the self-administration valve proposed in the present study is a good alternative to the routinely used methods in TUSPB since it is a safe, cost-effective technique that promotes analgesia on demand without the need of an anesthesiologist [9,10].

Nitrous oxide can be self-administered for analgesia in various procedures, such as intra-articular injection of drugs[11], vascular access puncture[12], sigmoidoscopy[13], colonoscopy[14], ophthalmologic procedures[15] and prostate biopsy[16]. Nitrous oxide has been used in emergencies, accident care and patient transport in ambulances[17].

Considering that pain is an event that has a socio-cultural influence[18], and to date, no study with these characteristics has been performed in the Brazilian population, this clinical trial is justified.

The hypothesis of the present study is that the inhalation of 50–50% N_2_O-O_2_, per self-administration valve, will be able to reduce pain in patients undergoing TUSPB.

The objective of the study was to evaluate the pain intensity in patients submitted to TUSPB. The secondary objectives were to determine the frequency of adverse events and the degree of satisfaction of these patients with the treatment proposed in the research.

## Material and methods

This prospective and randomized clinical trial was performed after approval by the Research Ethics Committee of the Antônio Pedro University Hospital (Hospital Universitário Antônio Pedro) of the Fluminense Federal University (Universidade Federal Fluminense–UFF) on 02/05/2015 (Presentation Certificate for Ethical Assessment (CAAE) n. 39144914.8.0000.5243) and was registered in the *Clinical Trials* on 14/09/2016 (NCT: 02899182), and the authors confirm that all ongoing and related trials for this intervention are registered.

A total of 84 men aged 18 years old or older who were ASA I to III and underwent elective or outpatient transrectal ultrasound-guided prostate biopsy were recruited, from May 2015 to November 2016.

The following exclusion criteria were adopted: patients who had participated in another study in the last month, those using psychoactive drugs, those known to be hypersensitive to any study medication, patients with severe diseases in organs such as the kidneys, liver, lungs, heart and brain, patients with impossibility to report the intensity of pain and those unable to inhale the gas mixture through the self-administering device.

An informed consent form was signed by each of the volunteer participants, who were advised of the risks and benefits of the research. The patients were divided into two groups according to a sequence of random numbers generated electronically through the program *GraphPad Prism®*. Forty-two patients were allocated to the O_2_ group (C) and 42 to the N_2_O oxide group (NO). Group C received topical anesthesia in the anal canal (lidocaine hydrochloride jelly 2%—CRISTÁLIA Produtos Químicos e Farmacêuticos, Itapira, SP, Brazil) plus 100% oxygen inhalation under a facemask. In turn, the NO group received topical anal anesthesia (lidocaine hydrochloride jelly 2% gel—CRISTÁLIA Produtos Químicos e Farmacêuticos, Itapira, SP, Brazil) plus inhalation of the 50–50% N_2_O-O_2_ gas mixture (LIVOPAN®, Linde Gases, Rio de Janeiro, RJ, Brazil) through a self-administration valve.

Patients were monitored in the procedure room using a noninvasive blood pressure device, electrocardioscope and pulse oximeter. The values of systolic blood pressure, diastolic blood pressure, heart rate and peripheral oxygen saturation were recorded immediately before and after the procedure.

The biopsies were performed by the same radiologist, with the patient in the left lateral decubitus position. A GE Logic S6 device (GE Healthcare, Waukesha, WI, USA) and a GE E8C RS probe were used to perform the transrectal ultrasonographies. The biopsies were obtained in an average of 10 punctures with a Gallini 18G x 25 cm needle.

An anesthesiologist followed the examinations without intervening in the analgesia proposed by randomization. Ten minutes after the examination was completed, an investigator who was not involved in the procedure presented and explained the 10 cm visual analog scale (VAS) to the patients, to evaluate their pain intensity (0 to 10) during the procedure and the level of satisfaction (0 to 10) with the administered treatment. The occurrence rates of nausea, vomiting, dizziness, hemodynamic changes, laughter crisis and somnolence during the examination were also evaluated.

The sample size was calculated based on the primary outcome, using the results of the study by Pita et al.^9^ as a parameter. Faced with a reduction of 30% in pain intensity when the 50–50% N_2_O-O_2_ mixture was used for analgesia, a sample of 42 patients in each group was required to detect such a difference, with respective probabilities of type-1 and type-2 error of 0.05 (α) and 0.2 (β) by the two-tailed test (study power of 80%).

Values were expressed as numbers of patients, means, medians, interquartile ranges and 95% confidence intervals. The Shapiro-Wilk test showed that all parameters had a normal distribution, which allowed the use of a parametric Student's t-test in the comparison of the NO group with the control group to calculate the two-tailed p-probability. A value of p < 0.05 was considered statistically significant. Statistical analysis was performed using SPSS software v.19.0 (IBM, New York, USA).

## Results

**Fig 1:**
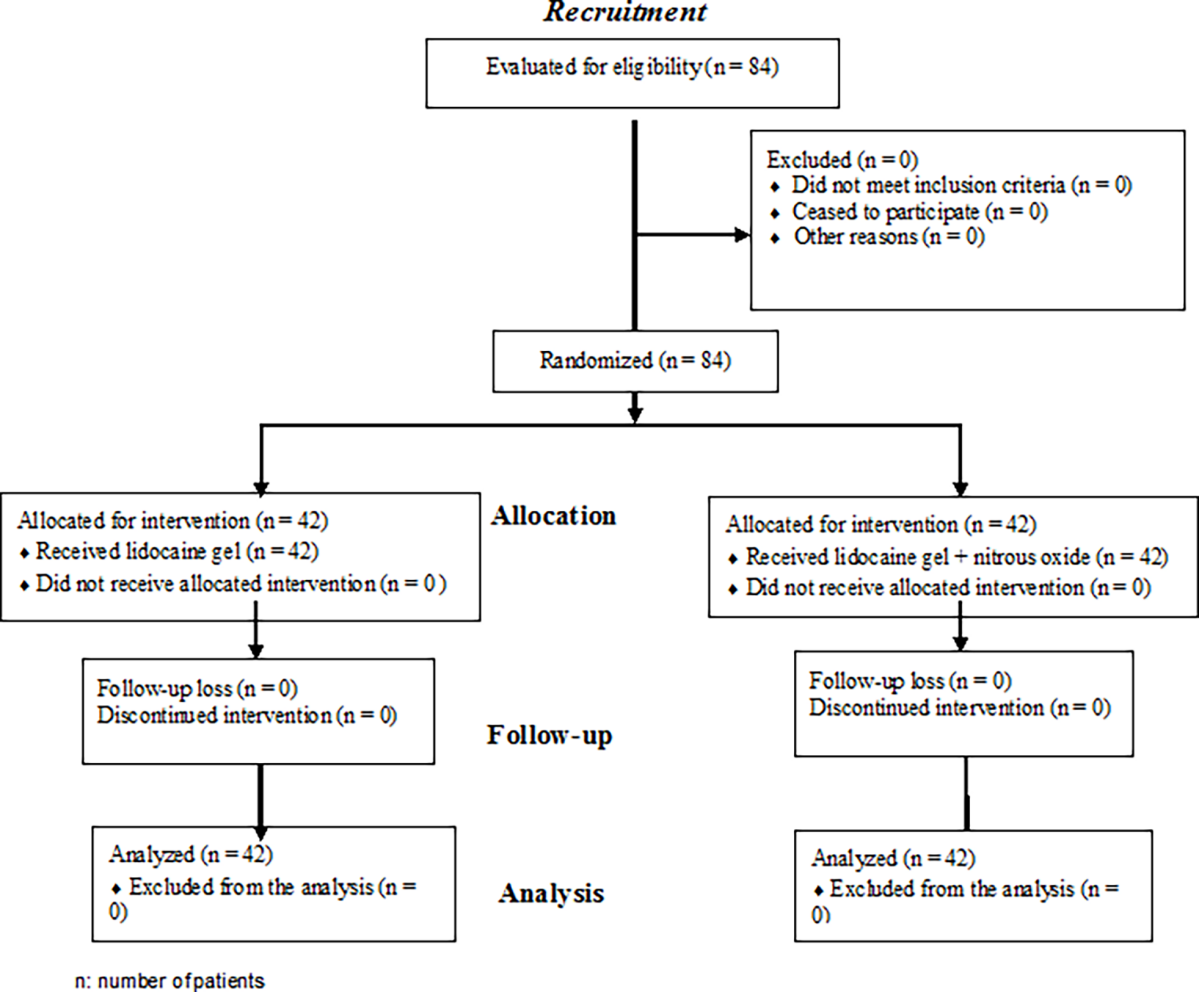
Flow chart of patients through the study.

We selected 84 eligible patients. (Fig 1)

There were no significant differences between the groups in relation to age, weight and height (Table 1).

**Table 1:** Demographic data for the nitrous oxide (NO) and control (C) groups.

Variables		n	Min	Percentile			Max		Mean	SD	95% CI		2-tailed p-value
				25%	Median	75%					Lower	Upper	
Age (year)	NO	42	53	63	70	76.5	86		69.45	8.42	66.83	72.08	0.0760
	C	42	52	62	65.5	72	83		66.38	7.19	64.14	68.62	
Weight (kg)	NO	42	46	64.5	70	77.5	106		70.5	12.06	66.74	74.26	0.3453
	C	42	50	65	73	80	115		73.05	12.53	69.14	76.95	
Height (m)	NO	42	1.58	1.65	1.695	1.72	1.82		1.688	0.059	1.67	1.71	0.9558
	C	42	1.5	1.65	1.7	1.725	1.8		1.689	0.0588	1.671	1.71	

Two-tailed p-value: Unpaired Student’s t-test; NO: Nitrous oxide; C: Control; kg: Kilograms; m: Meters; N: Number of patients.

Regarding pain during the procedure, the main evaluated outcome, 13 patients in the C group (30,9%) and only 3 patients (7,1%) in the NO group classified it as intense pain (odds ratio [OR] 0.172, 95% confidence interval [CI], two-tailed p = 0.012). Twenty-seven patients (64%) in the NO group and 8 patients (19%) in the C group classified pain as mild (OR 7.65, 95% CI, two-tailed p = 0.0001). Some patients classified the pain as moderate, including 21 patients in the C group (50%) and 12 patients (28,5%) in the NO group (OR 0.4, 95% CI, two-tailed p = 0.074).

The mean pain scores of the two groups were 5.95 (min = 0; max = 10) in the C group and 2.52 (min = 0; max = 10) in the NO group (Table 2) with a two-tailed p value of 0.0001.

**Table 2:** Pain intensity and patient contentedness in VAS values for the nitrous oxide (NO) and control (C) groups.

Variables		n	Min	Percentile			Max		Mean	SD	95% CI		2-tailed p-value
				25%	Median	75%					Lower	Upper	
Pain	NO	42	**0**	0	1.5	4	10	2.52		2.89	1.62	3.42	<0.0001
	C	42	0	4	6.5	8	10	5.95		2.69	5.12	6.79	
Contentedness	NO	42	0	1	5	8	9	8.14		1.26	8.1	8.9	<0.0001
	C	42	0	2.5	5	7	9	4.69		2.62	3.9	5.5	

VAS: Visual Analog Scale, N: Number of patients, two tailed P value: Unpaired Student’s t-test

Satisfaction indexes also differed between the two groups, with means of 8.14 (min = 1, max = 10) in the NO group and 4.69 (min = 0; max = 8) in the C group (Table 2) and a two-tailed p value of 0.0001.

In the C group, the frequencies of adverse events were as follows: 4 (9.5%) patients with somnolence; 3 (7,1%) with dizziness, 3 (7.1%) with euphoria and, finally, 5 (11.9%) with discomfort. In the NO group, the adverse events were: 4 (9.5%) patients experienced somnolenc; dizziness 4 (9.5%); vomiting 1 (2,3%); euphoria 5 (11,9%); discomfort 2 (4,7%) (Table 3).

**Table 3:** Adverse events.

Adverse events	Nitrous oxide n(*%*)	Control n(*%*)	P value (*)
Somnolence	**4** (9.5)	4 (9.5)	0.8182
Dizziness	4 (9.5)	3 (7.1)	
Nausea	0	0	
Vomiting	1 (2.3)	0	
Euphoria (laughter crisis)	5 (11.9)	3 (7.1)	
Discomfort	2 (4.7)	5 (11.9)	

(*) p-value (two tailed): Mann-Whitney nonparametric analysis for independent samples

There were no differences in systolic blood pressure, diastolic blood pressure, heart rate and peripheral oxygen saturation between the two studied groups either before or immediately after the procedure (Table 4).

**Table 4:** Systolic (SAP) and diastolic (DAP) arterial pressures, heart beat frequency (HBF) and peripheral oxygen saturation (SpO_2_) before (B) and after (A) prostate biopsy.

Variables			n	Min	Percentile			Max	Mean	SD	C. I. (95%)		2 tailed P value
					25%	Median	75%				Lower	Upper	
**B**	PAS (mm Hg)	NO	42	110	120	146	158.5	199	144.9	23.12	137.7	152.1	0.6648
		C	42	79	129.5	143	160	252	147.3	27.38	138.7	155.8	
	PAD (mm, Hg)	NO	42	63	73	80	88	109	81.64	12.09	77.87	85.41	0.3777
		C	42	53	74.5	84	91.5	119	84.12	13.45	79.93	88.31	
	FC (bpm)	NO	42	40	63.5	76	86.5	116	75.74	17.2	70.38	81.1	0.7375
		C	42	47	65	75	90	124	76.98	16.53	71.83	82.13	
	SpO_2_ (%)	NO	42	94	97	97	98	99	97.2	0.98	96.89	97.5	0.3920
		C	42	95	97	97	98	99	97.38	0.99	97.07	97.69	
**A**	PAS (mm Hg)	NO	42	104	130	144	166.5	249	148.8	28.98	139.8	157.8	0.5999
		C	42	73	128	144	160.5	246	145.5	28.63	136.6	154.4	
	PAD (mm, Hg)	NO	42	53	71.5	81.5	93	150	83.6	17.26	78.22	88.97	0.7908
		C	42	31	72.5	83	92	122	82.64	15.49	77.82	87.47	
	FC (bpm)	NO	42	45	68	84.5	94	127	82.17	16.19	77.12	87.21	0.3209
		C	42	46	64	75.5	89	120	78.45	17.86	72.89	84.02	
	SpO_2_ (%)	NO	42	93	97	98	98	99	97.44	1.097	97.09	97.79	0.5418
		C	42	95	97	97.5	98	98	97.31	0.81	97.06	97.56	

NO: Nitrous oxide; C: Control; bpm: Beats per minute; two-tailed p-value: Unpaired Student’s t-test; N: Number of patients.

## Discussion

The fixed gas mixture of 50% oxygen and 50% nitrous oxide has been safely used as an option for sedation and analgesia for various diagnostic or therapeutic procedures[11–17,19].

The pharmacological underpinnings for such indications are widely known[20,21]. Historical studies on potency have shown that 30% nitrous oxide is equivalent to 10–15 mg of morphine[22,23].

This is the first study performed in the Brazilian population using the fixed gas mixture of oxygen and nitrous oxide at 50% through a self-administration valve (Livopan®) in a tranrectal ultrasound-guided prostate biopsy.

Five to 95% of patients report pain or discomfort during transrectal prostate biopsy. The penetration of the prostatic capsule by the needle is the main cause of pain during the biopsy, and the degree of major discomfort occurs at the time of the introduction of the transducer [24]. Regarding intensity, a large number of patients report moderate and intense pain during TUSPB [25]. The innervation of the prostate originates in the lower hypogastric plexus, formed by sacral fibers S2 to S4 [26].

The present study demonstrated that, in the Brazilian population, the administration of the 50–50% N_2_O-O_2_ mixture through a self-administration valve was able to significantly reduce pain intensity in transrectal ultrasound-guided prostate biopsy.

In this study, the mean pain intensity of the patients in the NO group was 2.52, while the mean pain intensity of those in the C group was 5.95 (p < 0.0001). A similar result was found by Masood et al. [16], who found mean pain intensities (Entonox®) of 2.2 (± 1.52) in the nitrous oxide group and of 5.73 (± 1.62) in the control group (p < 0.001). McIntyre et al. [27] also achieved a significant reduction in pain scores of the nitrous oxide group (median 1.1) compared with the control (air) group (median 3.4) (p < 0.001). A different result was observed by Spie et al.[28], who did not find a significant difference, although pain tended to be lower in the nitrous oxide group (mean 2.9 in the nitrous oxide group and 3.5 in the control group, p = 0.10).

Similar to that found in the present study, Manikandan et al. [29] also found a mean pain intensity of 2.2 (± 1.59) when they used a mixture of oxygen and 50% nitrous oxide (Entonox®). However, the control group (no analgesia before prostate biopsy) had an average of 2.9 (± 1.59). Notably, even without any kind of analgesia, the control group of the study by Manikandan et al [29]. presented a low mean pain intensity compared with those in the present study and in the study by Masood et al. [16] This difference between the results of the control group of the present study and those of the study by Manikandan et al.[29] can be explained by differences in the timing of the assessment; in the study method of Manikandan et al.[29], the pain intensity assessment was performed immediately before and after the procedure, while in the present study, it was performed 10 minutes after the test.

Spie et al. [28] found lower pain intensity values in the control group (mean of 3.5) than those observed in the present study (mean 5.95) and the study by Masood et al.[16] (mean 5.73).

McIntyre et al.[27] also obtained median values for pain intensity of 3.4, similar to those of the study by Spie et al.[28] It should be noted that in the study by Spie et al.[28], the present study and the study by Masood et al.[16], intrarectal lidocaine gel was used in the control group, while in the study by Manikandan et al [29], no analgesia was used. This difference between the pain scores in the control groups could be explained by the possible differences between prostate size, number of punctures during biopsy, type of needle or technique employed [27]. It could also be explained by sociocultural differences among participants, who were French in the study by Spie et al.[28], British in the study by Masood et al.[16] and Manikandan et al.[29] and Brazilian in the present study. This hypothesis is justified in that the interindividual variation in pain intensity in response to an identical procedure, injury or noxious condition has been widely described. Sensitivity to pain is influenced by genetic factors, epigenetic factors, personal history and psychological factors. In addition, personal beliefs, pain representation and personal cultural experience can affect the intensity and expression of pain [18].

The present study demonstrated that patients in the nitrous oxide group had a significantly higher degree of satisfaction than those in the control group, with means of 8.14 (NO) vs 4.69 (C) (p < 0.0001). We have not found information regarding patient satisfaction with the type of analgesia received in previously published studies on nitrous oxide in transrectal ultrasound-guided prostate biopsy, which makes this result unprecedented. Ball et al.[30] studied the degree of satisfaction of patients with continuous or intermittent use of nitrous oxide and obtained a high satisfaction rate, with means of 9.9 (± 0.4) in the continuous group and 9.7 (± 0, 9) in the intermittent group (p = 0.23). Maleskar et al.[31] found a high degree of patient satisfaction (median of 9.4) when they received nitrous oxide for analgesia. We emphasize that in both the studies by Ball et al.[30] and by Maleskar et al.[31], the procedure studied was colonoscopy, different from that of the present study.

The frequency of adverse events was relatively low in our study. This result is similar to that found by McIntyre et al.[27], who demonstrated the absence of adverse effects, respiratory problems and prolonged drowsiness when nitrous oxide was used for prostate biopsy. Spie et al.[28], unlike the study by McIntyre et al.[27] and the present study, found higher frequencies of adverse effects. We could justify such differences for methodological reasons, especially the way data was collected, which in the research of Spie et al.[28] was performed immediately after the end of the procedure, and the patients responded to a questionnaire listing the adverse effects: anxiety, euphoria, excitement, restlessness, memory, changes in environmental and sensory perceptions, whereas in curent study, spontaneous reporting was utilized (assessing somnolence, nausea, vomiting, laughter crisis/euphoria, dizziness, discomfort) 10 minutes after the end of the procedure.

The present study has some limitations. The participants were all residents of the state of Rio de Janeiro and, due to the continental characteristics of the brazilian territory, there are regional differences in pain intensity even in Brazil, which limits the extrapolation for the entire brazilian population. The assessment of pain intensity alone is not able to determine the multidimensionalities that are part of the nature of pain. Another limitation of the study was due to late prostate biopsy being performed only in men, the results can not be extrapolated to women and to other diagnostic or therapeutic procedures.

In the current study, there were no significant differences in the values of systolic blood pressure, diastolic blood pressure, heart rate and peripheral O_2_ saturation between the two groups at the studied moments. These results point to the hemodynamic stability presented by patients in the nitrous oxide group. The same results were found by Massod et al.[16], who did not find significant differences in heart rate between nitrous oxide and control groups, although the mean value for heart rate was lower than that in the nitrous oxide group, which could suggest better analgesia. Massod et al.[16] also found no significant difference in oxygen saturation, a result similar in our study.

## Conclusion

The present study concluded that pain intensity was significantly reduced in patients who inhaled N_2_O:O_2_ (50–50%) by means of a self-administered valve for transrectal ultrasound-guided prostate biopsy. The frequency of adverse effects and systolic and diastolic blood pressures, heart rate and peripheral oxygen saturation values were similar between those who inhaled and those who did not inhale nitrous oxide. The level of patient satisfaction was significantly higher among those who used the N_2_O:O_2_ mixture than among those who used oxygen alone.

## Supporting information

S1 File TCLE. (DOCX)

S2 File TCLE ingles. (DOCX)

S3 File Questionnaire—Original version. (DOCX)

S4 File Questionnaire—English. (DOCX)

S5 File CONSORT Plos One. (DOC)

S6 File Study protocol—Original version. (DOCX)

S7 File Study protocol—English. (DOCX)
