# Supplementary material for: A 50-50% mixture of nitrous oxide-oxygen in transrectal ultrasound-guided prostate biopsy: A randomized and prospective clinical trial
Source: PLoS One. 2018 Apr 27;13(4):e0195574. doi: 10.1371/journal.pone.0195574 (PMC5922537; doi:10.1371/journal.pone.0195574)
Supplement: S1 File — (DOCX) [file pone.0195574.s001.docx]

**TERMO DE CONSENTIMENTO LIVRE E ESCLARECIDO**

**(em 2 vias)**

**TÍTULO DO PROJETO:** “Mistura óxido nitroso-oxigênio (50-50%) em biópsia de próstata”

**RESPONSÁVEIS PELO PROJETO:** Dr. Gabriel Cazarim

**ORIENTADORES RESPONSÁVEIS:** Dr. Ismar Lima Cavalcanti, Dra. Núbia Verçosa

**UNIVERSIDADE FEDERAL FLUMINENSE**

**HOSPITAL UNIVERSITÁRIO ANTÔNIO PEDRO**

**SERVIÇO DE RADIOLOGIA E DE ANESTESIOLOGIA**

Telefones para contato: (___) ______________ Nº do Termo: _____________

Nome do voluntário: _______________________________________________________

Idade: _______ CPF ______________________ R.G. ____________________________

Responsável legal (se for o caso): ____________________________________________

R.G. Responsável legal: ____________________________________________________

O senhor está sendo convidado a participar do projeto de pesquisa “Mistura óxido nitroso-oxigênio (50-50%) em biópsia de próstata”, de responsabilidade dos médicos pesquisadores Gabriel Cazarim e Hugo Siqueira. O objetivo principal desta pesquisa é avaliar a capacidade do *óxido nitroso* de reduzir o desconforto/dor de pacientes submetidos à biópsia de próstata por via retal. O senhor será orientado a respirar um gás ou uma mistura de gases durante o procedimento e, logo depois, a responder um questionário.

**Justificativa:**

Apesar de bem tolerado por muitos pacientes, cerca de 65 a 90% dos homens submetidos à biópsia de próstata via retal se queixam de desconforto, associado ou não a dor. Para melhor tolerância, alguns métodos de analgesia/sedação foram propostos, como o uso da mistura óxido nitroso-oxigênio (50-50%).

**Objetivos:**

Reduzir o desconforto dos pacientes submetidos à biópsia de próstata, ajudando a realização do exame.

**Procedimentos:**

- Avaliação pré-anestésica.
- Monitorização padrão (Resolução CFM n° 1.802/06): pressão arterial, frequência cardíaca e oxigenação sanguínea.
- Inalação do gás indicado pelo pesquisador durante a biópsia.
- Aplicação do questionário após o procedimento.
- Possibilidade de inclusão no grupo controle (oxigênio puro) ou experimental (mistura óxido nitroso-oxigênio), sem tomar conhecimento de qual grupo faz parte.

**Benefício(s) esperado(s):**

Redução da intensidade da dor/desconforto durante o procedimento.

**Responsabilidades ao aceitar participar do estudo:**

- Seguir as instruções dadas pelo pesquisador;
- Respirar o gás indicado pelo pesquisador durante a biópsia de próstata;
- Responder o questionário após o procedimento.

**Riscos:**

O senhor está sendo informado que o óxido nitroso é um agente anestésico que tem sido usado por décadas com segurança. A substância, na concentração apresentada, produz analgesia e sedação. Além dos riscos já previstos do ato cirúrgico, incluem-se:

- euforia;
- tonteiras;
- náuseas e vômitos.

Todos os efeitos do medicamento são passageiros.

**Obrigações:**

O senhor não é obrigado a participar deste estudo. Se assim desejar, simplesmente informe isto ao profissional responsável. Poderá retirar seu consentimento a qualquer momento e deixar de participar do estudo, sem que isto traga qualquer prejuízo, bastando para isso comunicar sua decisão ao médico.

A qualquer momento, poderá obter outras informações acerca do estudo, ainda que isto possa afetar sua vontade de participar do mesmo. Serão fornecidos respostas ou esclarecimentos a qualquer dúvida acerca dos procedimentos, riscos, benefícios e outros assuntos relacionados com a pesquisa.

**Compensação:**

Havendo comprovação de que tenha ocorrido danos diretamente causados pela pesquisa, terá direito a tratamento médico na referida instituição, com as despesas médicas extras não disponíveis na unidade pagas pela equipe do estudo.

**Confidencialidade:**

Os registros de cada paciente serão feitos através de um número de identificação e das iniciais do paciente. As informações obtidas com o estudo serão confidenciais e utilizadas apenas com o propósito científico. As autoridades sanitárias e os Comitês de Ética podem verificar os registros do estudo, e não haverá nenhuma liberação ou publicação destes registros que revelem sua identidade.

**Término do estudo:**

A equipe médica pode interromper sua participação neste estudo a qualquer momento, se julgar apropriado devido ao descumprimento dos procedimentos solicitados, ou no seu melhor interesse.

**Consentimento:**

Eu compreendo que estou permitindo minha participação voluntária neste estudo. Posso retirar-me a qualquer momento sem qualquer prejuízo de benefícios a que tenho direito. Qualquer dúvida que eu tenha em relação a qualquer aspecto deste estudo ou sobre meus direitos, será respondida por:

Dr. Gabriel Cazarim – Serviço de Anestesiologia/HUAP – Telefone: (21) 99308-0393

Eu, ________________________________________________ concordo em participar do estudo acima e compreendo que minha recusa em participar, ou retirada posterior do estudo, não trará prejuízos de qualquer natureza.

Niterói,_______de_________________de_________

_______________________________­­­­­­­ _______________________________

Voluntário Médico

_______________________________ ________________________________

Testemunha Testemunha
