# Supplementary material for: A 50-50% mixture of nitrous oxide-oxygen in transrectal ultrasound-guided prostate biopsy: A randomized and prospective clinical trial
Source: PLoS One. 2018 Apr 27;13(4):e0195574. doi: 10.1371/journal.pone.0195574 (PMC5922537; doi:10.1371/journal.pone.0195574)
Supplement: S2 File — (DOCX) [file pone.0195574.s002.docx]

**CONSENT TERM**

**TITLE OF THE PROJECT:** A 50-50% mixture of nitrous oxide-oxygen in transrectal ultrasound-guided prostate biopsy: a randomized and prospective clinical trial.

**RESPONSIBLE FOR THE PROJECT: Dr.** Gabriel Cazarim, MD.

**RESPONSIBLE GUIDELINES:** PhD. Ismar Lima Cavalcanti, PhD. Núbia Verçosa

**FEDERAL FLUMINENSE UNIVERSITY**

**ANTÔNIO PEDRO UNIVERSITY HOSPITAL**

**RADIOLOGY AND ANESTHESIOLOGY SERVICE**

Phone: (___) ______________ number: _____________

Name: _______________________________________________________

Age: _______ ID ______________________ ID ____________________________

Legal person (if applicable): ____________________________________________

You have been invited to participate in the research project: " A 50-50% mixture of nitrous oxide-oxygen in transrectal ultrasound-guided prostate biopsy: a randomized and prospective clinical trial.", The responsible of the research is Gabriel Cazarim, mD. The main objective of this research is to evaluate the ability of nitrous oxide to reduce the discomfort / pain of patients undergoing rectal prostate biopsy.
 You will be instructed to breathe a gas or a mixture of gases during the procedure and then respond to a questionnaire.

**Justification:**

Although well tolerated by many patients, about 65-90% of men undergoing rectal prostate biopsy complain of discomfort, associated or not with pain. For better tolerance, some analgesia / sedation methods have been proposed, such as the use of the nitrous oxide-oxygen mixture (50-50%).

**Goals:**

Reduce the discomfort of patients undergoing prostate biopsy, helping to perform the test.

**Procedures:**

• Pre-anesthetic evaluation.

• Standard monitoring (CFM Resolution 1,802 / 06): blood pressure, heart rate and blood oxygenation.

• Inhalation of the gas indicated by the investigator during the biopsy.

• Application of the questionnaire after the procedure.

• Possibility of inclusion in the control group (pure oxygen) or experimental (mixture of nitrous oxide-oxygen), without knowing which group is involved.

**Expected benefit (s):**

Reduction of pain intensity / discomfort during the procedure.

**Responsibilities in accepting to participate in the study:**

• Follow the instructions given by the researcher;

• Breathe the gas indicated by the researcher during the prostate biopsy;

• Respond to the questionnaire after the procedure

**Risks:**

You are being informed that nitrous oxide is an anesthetic agent that has been safely used for decades. The substance, in the presented concentration, produces analgesia and sedation. In addition to the risks already foreseen in the surgical procedure, the following are included:

• euphoria;

• dizziness;

• nausea and vomiting.

All effects of the medicine are transient.

**Obligations:**

You are not obliged to participate in this study. If you wish, simply inform the responsible professional. You may withdraw your consent at any time and cease to participate in the study, without this causing any damage, simply by communicating your decision to the doctor.

At any time, you may obtain further information about the study, although this may affect your willingness to participate. Answers or clarifications will be provided to any questions about the procedures, risks, benefits and other matters related to the research.

**Compensation:**

If there is evidence that damage has been directly caused by the research, you will be entitled to medical treatment at the institution, with the extra medical expenses not available in the unit paid by the study team.

**Confidentiality:**

The records of each patient will be made through an identification number and the initials of the patient. The information obtained from the study will be confidential and used only for scientific purposes. Health authorities and Ethics Committees can verify the study records, and there will be no release or publication of these records revealing their identity.

**End of study:**

Medical staff may discontinue your participation in this study at any time if you deem it appropriate due to noncompliance with the requested procedures, or in your best interest.

**Consent:**

I understand that I am allowing my voluntary participation in this study. I can withdraw at any time without any loss of benefits to which I am entitled. Any questions I have about any aspect of this study or about my rights will be answered by:

Dr. Gabriel Cazarim, MD - Anesthesiology Service /HUAP – PHONE: (21) 99308-0393

I, ________________________________________________ agree to participate in the above study and understand that my refusal to participate, or subsequent withdrawal of the study, will not result in any loss whatsoever.

Niterói,_______ __________________________

_______________________________­­­­­­­ _______________________________

volunteer doctor

_______________________________ ________________________________

attestant attestant
