# Supplementary material for: A 50-50% mixture of nitrous oxide-oxygen in transrectal ultrasound-guided prostate biopsy: A randomized and prospective clinical trial
Source: PLoS One. 2018 Apr 27;13(4):e0195574. doi: 10.1371/journal.pone.0195574 (PMC5922537; doi:10.1371/journal.pone.0195574)
Supplement: S3 File — (DOCX) [file pone.0195574.s003.docx]

Questionário – LIVOPAN

**IDENTIFICAÇÃO:**

Nome: _________________________________________________ Idade: ______ anos

Prontuário: _____________ Cor: ___________ Peso: ______ Kg Altura: _______ m

Pesquisador: _____________________________ Sujeito: ______ Data: ___ /___ /___

**COMORBIDADES:**

( ) HAS ( ) DM ( ) DAC ( ) dislipidemia ( ) AVC ( ) demência ( ) IRC ( ) asma

( ) outros: ______________________________________________________________________

( ) tabagista ( ) ex-tabagista maços-anos: ____________ ( ) etilista ( ) ex-etilista

Medicações: ______________________________________________________________________

**EXAMES LABORATORIAIS:**

| Ht (%): |  | Cr: |  |
| --- | --- | --- | --- |
| Ht: |  | Uréia: |  |
| Leucócitos: |  | PSA total: |  |
| Plaquetas: |  | PSA livre: |  |
| Outros: |  | PSA relação: |  |

Data dos exames: ___ /___ /___

**SINAIS VITAIS:**

INICIAL FINAL

| PA: |  |  |
| --- | --- | --- |
| FC: |  |  |
| SpO_2_: |  |  |

**QUESTIONÁRIO:**

| SIM | NÃO |
| --- | --- |
| SIM | NÃO |

1. Foi a primeira vez que realizou o exame?

O senhor sentiu dor ao realizar o exame?

1. Qual a intensidade da dor? NOTA: _______


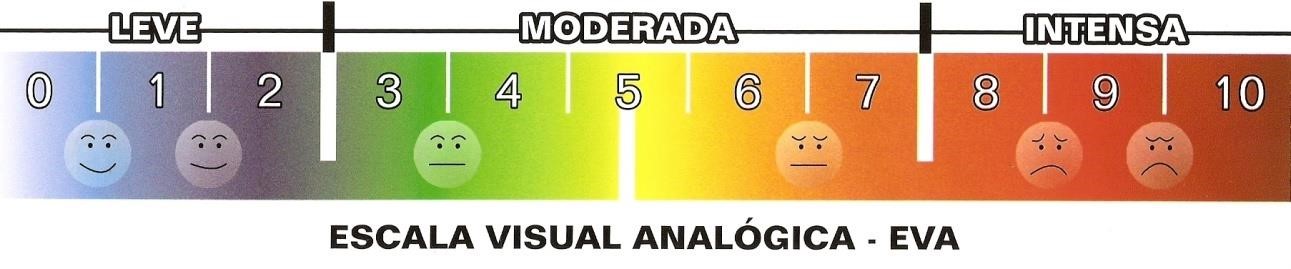


| SIM | NÃO |
| --- | --- |

1. O senhor ficou satisfeito com o resultado da anestesia?

O senhor apresentou:

| SIM | NÃO |
| --- | --- |
| SIM | NÃO |
| SIM | NÃO |
| SIM | NÃO |
| SIM | NÃO |

Sono?

Tonteiras?

Náuseas?

Vômitos?

Crise de riso?

| SIM | NÃO |
| --- | --- |
| SIM | NÃO |

Sentiu-se alegre?

Mal estar?
