# Supplementary material for: A 50-50% mixture of nitrous oxide-oxygen in transrectal ultrasound-guided prostate biopsy: A randomized and prospective clinical trial
Source: PLoS One. 2018 Apr 27;13(4):e0195574. doi: 10.1371/journal.pone.0195574 (PMC5922537; doi:10.1371/journal.pone.0195574)
Supplement: S4 File — (DOCX) [file pone.0195574.s004.docx]

Questionnaire – LIVOPAN

**IDENTIFICATION:**

Name: _________________________________________________ Age: ______ years

Medical Record: _____________ Color: ___________ Weight: ______ Kg Height: _______ m

Researcher: _____________________________ Date: ___ / ___ / ___

**COMORBITIES:**

( ) HYPERTENSION ( ) DIABETES ( ) CORONARY DISORDER ( ) DISLIPIDEMIA ( ) STROKE ( ) DEMENTIA ( ) RENAL DISEASE ( ) ASTHMA ( ) OTHERS:____________________________________

( ) SMOKER ( ) EX-SMOKER ( ) DRINKER

Medications: ______________________________________________________________________

**VITAL SIGNS:**

INICIAL FINAL

| BP: |  |  |
| --- | --- | --- |
| HR: |  |  |
| SpO_2_: |  |  |

**QUESTIONNAIRE:**

| YES | NO |
| --- | --- |
| YES | NO |

1. Was this the first time you took the exam?
2. Did you feel pain when taking the exam?

How intense was the pain? GRADE: _______


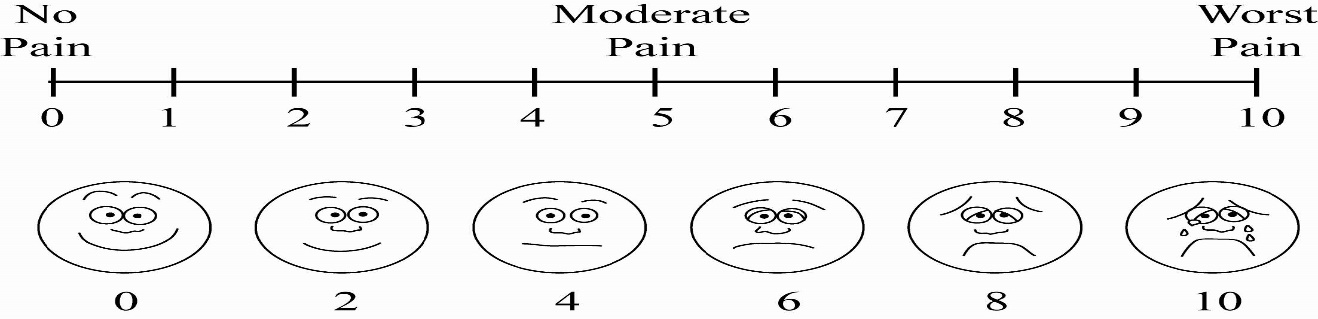


| YES | NO |
| --- | --- |

1. Were you satisfied with the result of the anesthesia?

1. Did you feel:

| YES | NO |
| --- | --- |
| YES | NO |
| YES | NO |
| YES | NO |
| YES | NO |
| YES | NO |
| YES | NO |

sleepiness?

dizziness?

nausea?

spew?

laugh?

happiness?

malaise?
