# Supplementary material for: A 50-50% mixture of nitrous oxide-oxygen in transrectal ultrasound-guided prostate biopsy: A randomized and prospective clinical trial
Source: PLoS One. 2018 Apr 27;13(4):e0195574. doi: 10.1371/journal.pone.0195574 (PMC5922537; doi:10.1371/journal.pone.0195574)
Supplement: S6 File — (DOCX) [file pone.0195574.s006.docx]

**UNIVERSIDADE FEDERAL FLUMINENSE**

**HOSPITAL UNIVERSITÁRIO ANTÔNIO PEDRO**

**SERVIÇO DE ANESTESIOLOGIA**

**SERVIÇO DE RADIOLOGIA**

Mistura Óxido Nitroso-Oxigênio (50-50%) em Biópsia de Próstata Transretal Guiada por Ultrassonografia

PROTOCOLO DE PESQUISA

(Resolução CNS n° 466/12)

**PESQUISADORES:**

Dr. Gabriel da Silva Cazarim

**ORIENTADORES:**

Dr. Ismar Lima Cavalcanti

Dr. Núbia Verçosa Figueiredo

**NITERÓI/RJ**

**2014**

Mistura Óxido Nitroso-Oxigênio (50-50%) em Biópsia de Próstata Transretal Guiada por Ultrassonografia

_________________________________

Dr. Gabriel da Silva Cazarim

CRM 52-94.802-0

CPF 124.666.487-92

Celular: (021) 99308-0393

e-mail: gabriel_cazarim@hotmail.com

com autorização de:

_________________________________

Dr. Haberlandh Sodré Lima

Diretor Médico do HUAP

_________________________________

Dr. Nisval de Magalhães Júnior

Chefe do SEAN/Centro Cirúrgico do HUAP

_________________________________

Dr. Alair Sarmet Santos

Chefe do Serviço de Radiologia

SUMÁRIO

1 INTRODUÇÃO 4

2 DESCRIÇÃO DA PESQUISA 4

2.1 DESCRIÇÃO DOS PROPÓSITOS E DAS HIPÓTESES A SEREM TESTADAS 4

2.2 ANTECEDENTES CIENTÍFICOS E DADOS QUE JUSTIFIQUEM A PESQUISA 4

2.3 DESCRIÇÃO DETALHADA E ORDENADA DO PROJETO DE PESQUISA 4

2.3.1 MATERIAIS E MÉTODOS 5

2.3.2 SELEÇÃO, RANDOMIZAÇÃO E CEGAMENTO DE PACIENTES 5

2.3.3 MEDIDAS DE DESFECHO 5

2.3.4 CASUÍSTICA. 5

2.3.5 REGISTRO E APRESENTAÇÃO DE DADOS 6

2.3.6 ANÁLISE ESTATÍSTICA 6

2.3.7 RESULTADOS ESPERADOS 6

2.3.8 REFERÊNCIAS 6

2.4 ANÁLISE CRITÍCA DE RISCOS E BENEFÍCIOS 7

2.5 DURAÇÃO TOTAL DA PESQUISA (A PARTIR DA APROVAÇÃO) 7

2.6 DAS RESPOSNSABILIDADES 8

2.6.1 DO PESQUISADOR 8

2.6.2 DA INSTITUIÇÃO 9

2.6.3 DO PATROCINADOR 9

2.7 DOS CRITÉRIOS PARA SUSPENDER OU ENCERRAR A PESQUISA 9

2.8 LOCAL DA PESQUISA 9

2.9 INFRAESTRUTURA NECESSÁRIA AO DESENVOLVIMENTO DA PESQUISA 9

2.10 ORÇAMENTO FINANCEIRO 10

2.11 PROPRIEDADE DAS INFORMAÇÕES GERADAS PELA PESQUISA 10

2.12 PUBLICAÇÃO DOS RESULTADOS 10

2.13 USO E DESTINAÇÃO DO MATERIAL E/OU DADOS COLETADOS 10

3 INFORMAÇÕES RELATIVAS AO SUJEITO DA PESQUISA 10

3.1 POPULAÇÃO A SER ESTUDADA 11

3.2 CRITÉRIOS DE EXCLUSÃO DE INDIVÍDUOS 11

3.3 PLANOS PARA RECRUTAMENTO E PROCEDIMENTOS 11

3.4 TERMO DE CONSENTIMENTO DO PACIENTE 11

# 1 INTRODUÇÃO

A biópsia prostática transretal guiada por ultrassonografia (BPTU), associada à dosagem plasmática do antígeno prostático específico (PSA) é o método padrão utilizado para diagnóstico precoce do câncer de próstata. Apesar de bem tolerado por muitos pacientes, 65 a 90%* dos homens submetidos à BPTU se queixam de desconforto, associado ou não a dor.

Diversos métodos de analgesia e sedação foram propostos: bloqueios nervosos peri ou intraprostático, anestesia tópica com lidocaína ou mistura eutética de anestésico local (EMLA) no local da punção, anestesia geral com propofol e remifentanil, entre outras técnicas*. Desta forma, a inalação de N_2_O-O_2_ (50–50%) por válvula de auto demanda pode ser uma boa alternativa aos métodos invasivos por ser uma técnica segura*, que promove analgesia sob demanda, de maneira a aumentar a tolerância a procedimentos dolorosos como BPTU, prescindindo da presença de anestesiologista.

# 2 DESCRIÇÃO DA PESQUISA

## 2.1 DESCRIÇÃO DOS PROPÓSITOS E DAS HIPÓTESES A SEREM TESTADAS

A hipótese da presente pesquisa é de que a auto-inalação da mistura Óxido Nitroso-Oxigênio (50-50%) diminui a intensidade da dor do paciente submetido à biópsia de próstata via transretal guiada por ultrassonografia.

Objetivo primário:

- Avaliar a intensidade da dor;

Objetivos secundários:

- Avaliar o grau de satisfação
- Medir a incidência de eventos adversos (náuseas e vômitos, sonolência, crise de riso, tonteira, euforia ou algum mal estar)
- Avaliar alterações hemodinâmicas.

Soma-se a possibilidade de futura padronização no HUAP.

## 2.2 ANTECEDENTES CIENTÍFICOS E DADOS QUE JUSTIFIQUEM A PESQUISA

O óxido nitroso (N_2_O) pode ser auto-administrado para analgesia em diversos procedimentos como analgesia de parto (9), injeções intra articulares de drogas (11), punção de acesso vascular (12), retossigmoidoscopia e colonoscopia (13, 14), procedimentos oftalmológicos (15) e biopsia de próstata (16). Na Europa, Entonox, como é chamada a mistura (N_2_O-O_2_ 50-50%), é utilizado em emergências no atendimento de acidentes e transporte em ambulâncias (17).

## 2.3 DESCRIÇÃO DETALHADA E ORDENADA DO PROJETO DE PESQUISA

### 2.3.1 MATERIAIS E MÉTODOS

O ensaio clínico será realizado no Hospital Universitário Antônio Pedro (HUAP), Niterói, RJ. Nos dias atuais, o procedimento é realizado nos ambulatórios do Hospital, sem anestesista.

O termo de consentimento livre e esclarecido (TCLE) será apresentado e assinado, em regime ambulatorial, por cada um dos participantes voluntários, que serão orientados acerca dos riscos e benefícios da pesquisa. Serão estudados 84 pacientes submetidos à biópsia de próstata via transretal guiada por ultrassonografia em regime ambulatorial no HUAP, distribuídos aleatoriamente em dois grupos de 42. O grupo controle (C) receberá anestesia local no sítio de punção, técnica padrão utilizada rotineiramente no HUAP para esse procedimento, mais inalação de oxigênio a 100% sob máscara facial. O grupo ON receberá anestesia local no sítio de punção mais inalação de mistura de N2O-O2 por válvula de auto-demanda.

Um médico anestesiologista acompanhará os exames respeitando a resolução do Conselho Federal de Medicina (CFM) N° 1.802/2006, sem poder intervir na analgesia proposta pela randomização. Uma escala visual analógica (EVA) de dor 0-10 e de satisfação 0-10 serão apresentados aos pacientes antes do procedimento e respondidos após o mesmo. O grupo que receberá a mistura de N_2_O-O_2_ será avaliado em relação a incidência de náuseas, vômitos, tonteiras, alterações hemodinâmicas, crise de riso, sonolência durante o exame.

### 2.3.2 SELEÇÃO, RANDOMIZAÇÃO E CEGAMENTO DE PACIENTES

Serão selecionados todos os pacientes que forem indicados à BPTU no HUAP no período de realização da pesquisa, à exceção daqueles os quais se encaixem nos critérios de exclusão. Pacientes serão aleatoriamente alocados em um dos dois grupos de técnica anestésica, de acordo com sequência de números aleatórios gerada eletronicamente. Por se tratar de estudo duplo cego, pacientes e pesquisador executante do questionário não saberão a qual grupo randomizado.

### 2.3.3 MEDIDAS DE DESFECHO

Como desfecho primário, diminuição da frequência e intensidade da dor durante a BPTU. Como desfecho secundário, determinacão da incidência de efeitos adversos e alterações hemodinâmicas, além do conhecimento do grau de satisfação dos pacientes com a analgesia realizada.

### 2.3.4 CASUÍSTICA.

Tomando-se como desfecho primário uma diferença de 30% (de 80 para 50%) no escore de dor classificada como intensa (EVA > 7) referida pelos pacientes após a realização da BPTU, um nível de significância estatística de 5% (α = 0,05) e um poder de teste de 80% (erro β = 0,20), 2 grupos de 38 pacientes fez-se necessário. Prevendo-se censuras nos dados obtidos, aumentamos a amostra em 10%, resultando na formação de 2 grupos de igual tamanho com 42 pacientes cada (n = 84). Um valor de p < 0,05 será considerado estatisticamente significativo.

### 2.3.5 REGISTRO E APRESENTAÇÃO DE DADOS

Os dados gerados pela pesquisa serão registrados e apresentados em documento próprio (anexos II, III e IV), de posse de toda equipe pesquisadora, salvo a “Ficha de Cegamento e Aleatorização” (anexo IV) cuja posse será de exclusividade do pesquisador piloto. Esses dados servirão de base para análise estatística de todo o trabalho.

### 2.3.6 ANÁLISE ESTATÍSTICA

Ao término da fase experimental e de coleta de dados, toda informação gerada pelo ensaio será submetida à análise estatística no SPSS v.19.0 (IBM, New York, USA). Os valores serão expressos em médias, medianas ou número de pacientes. O padrão de distribuição de normalidade dos dados será testado com o método de Shapiro-Wilk. Dados paramétricos serão analisados usando o teste T de Student para comparar a significância de médias entre os grupos. Dados não paramétricos serão comparados usando o teste de Wilcoxon entre os grupos . Um valor de p < 0,05 será considerado estatisticamente significativo.

### 2.3.7 RESULTADOS ESPERADOS

A proposta do ensaio clínico é a redução na intensidade da dor e aumento do conforto e índice de satisfação durante a BPTU, na vigência de baixa taxa de eventos adversos.

### 2.3.8 REFERÊNCIAS

1. Uno, H; Nakano, M; Ehara, H; Degushi, T (2008) *Indications for 14-core transrectal ultrasound-guided prostate biopsy*. Urology 71: (1) 23-27
2. Inal, G; Yazici, S; Adsan, O; Ozturk, B; Kosan, M; Ctinkaya, M. (2009) *Effect of periprostatic nerve blockade before transrectal ultrasound-guided prostate biopsy on patient comfort: A randomized placebo controlled study.* International Journal of Urology 11: 148-151
3. Ozveri, H; Cevik, I; Dillioglugil, O; Akdas, A. (2003) *Transrectal periprostatic lidocaine injection anesthesia for transrectal prostate biopsy: a prospective study* Prostate Cancer and Prostatic 6: 311-314
4. Autorino, R; de Sio, M; di Lorenzo, G; Damiano, R; Perdona, S; Cindolo, L; D'Armiento, M. (2005) *How to decrease pain dureing transrectal ultrasound guided prostate biopsy: a look at the literature* The Journal of Urology 174: 2091-2097
5. Kubo, Y; Kawakami, S; Numao, N; Takazawa, R; Fujii, Y; Masuda, H; Tsujii, T; Kihara, K. (2009) *Simple and effective local anesthesia for transperineal extended prostate biopsy: Application to three-dimensional 26-core biopsy* International Journal of Urology, 16: 420-423
6. Kang, SG; Tae, BS; Min, SH; Ko, YH; Kang, SH; Lee, JG; Kim, JJ; Cheon, J. (2011) *Efficacy and cost analysis of transrectal ultrasound-guided prostate biopsy under monitored anesthesia* Asian Journal of Andrology 13: 724-727
7. Bingqian, L; Peihuan, L; Yudong, W; Jinxing, W; Zhiyong, W. (2009) *Intraprostatic Local Anesthesia With Periprostatic Nerve Block for Transrectal Ultrasound Guided Prostate Biopsy.* The Journal of Urology 182: 479-484
8. Basar, H; Murad Basar, M; Ozan, S; Akpinar, S; Basar, H; Batislam, (2005) E. *Local anesthesia in trasnrectl ultrasound-guided prostate biopsy: EMLA cream as a new alternative tecnique.* Scandinavian Journal of Urology and Nephrology 39: 130-134
9. Pita, CP; Pazmino, S ; Vallejo, M; Pousada, DS ; Luis Hidalgo,L; Pérez-López, FR;Chedraui, P. (2012) *Inhaled intrapartum analgesia using a 50–50 %mixture of nitrous oxide–oxygen in a low-income hospital setting*; Archives of Gynecology and Obstettrics 10: 1007
10. Emmanouil, DE; Quock, RM (2007) *Advances in Understanding the Actions of Nitrous Oxide;* Anesthesia Progress 54: 9–18
11. Cleary AG, Ramanan AV, Baildam E, Birch A, Sills JA, Davidson JE. (2002) *Nitrous oxide analgesia during intra-articular injection for juvenile idiopathic arthritis*. Archives of Diseases in Children*.*  86: 416–418.
12. Gerhardt RT, King KM, Wiegert RS. (2001) *Inhaled nitrousoxide versus placebo as an analgesic and anxiolytic adjunct toperipheral intravenous cannulation*. American Journal of Emergency Medicine. 19: 492–494.
13. Harding TA, Gibson JA. (2000) *The use of inhaled nitrous oxide for flexible sigmoidoscopy: a placebo-controlled trial*. Endoscopy*.* 32: 457–460.
14. Forbes GM, Collins BJ*.* (2000) *Nitrous oxide for colonoscopy: a randomized controlled study.* Gastrointestinal Endoscopy*.* 51: 271–277.
15. Cook HL, Newsom RS, Mensah E, Saeed M, James D, Ffytche TJ. (2002) *Entonox as an analgesic agent during panretinalphotocoagulation*. Brazilian Journal of Ophtalmology 86: 1107–1108.
16. Masood J, Shah N, Lanes T, Andrews H, Simpson P, Barua JM. (2002) *Nitrous oxide (entonox) inhalation and tolerance oftransrectal ultrasound guided prostate biopsy: a double-blind randomized controlled study*. Journal of Urology 168: 116–120.
17. Baskett PJ. (1970) *Use of Entonox in the ambulance service*. Brazilian Medical Journal 2: 41–43.

## 2.4 ANÁLISE CRITÍCA DE RISCOS E BENEFÍCIOS

O uso da mistura óxido nitroso-oxigênio (50-50%) em anestesia oferece poucos efeitos colaterais aos pacientes, sendo os mais conhecidos: sedação, tonteiras. náuseas, e euforia. Em contrapartida, oferece boa qualidade analgésicas, apresentando-se como boa escolha para procedimentos ambulatoriais.

Além disso, os pacientes do grupo C e grupo ON, estão expostos ao riscos do próprio procedimento, sendo estes: hemospermia, hematúria, sangramento retal, infecção trato urinário e prostatite.

## 2.5 DURAÇÃO TOTAL DA PESQUISA (A PARTIR DA APROVAÇÃO)

Com o início da fase de coleta de dados (BPTU) em fevereiro/2015 e previsão de 4 (quatro) exames por semana devido a grande demanda, a duração da pesquisa deverá ser de 12 (doze) meses, podendo se estender por mais 6 (seis) meses. Toda a fase de coleta de dados deverá se encerrar ao término do primeiro semestre de 2015, restando apenas o processamento das amostras e análise estatística.

## 2.6 DAS RESPONSABILIDADES

### 2.6.1 DO PESQUISADOR

**RESPONSÁVEIS PELO ENSAIO / PESQUISADORES PRINCIPAIS**

**NOME**: Gabriel da Silva Cazarim, MD.

**UNIDADE:** Serviço de anestesiologia do HUAP-UFF

**CATEGORIA FUNCIONAL / REGIME DE TRABALHO:** médico residente/especializando em anestesiologia; 60 horas/semana.

**RESPONSABILIDADES:** seleção e aleatorização da amostra; administração do(s) gás(es) testado(s)

**NOME:** Hugo Siqueira, MD.

**UNIDADE:** Serviço de anestesiologia do HUAP-UFF

**CATEGORIA FUNCIONAL / REGIME DE TRABALHO:** médico residente/especializando em anestesiologia; 60 horas/semana.

**RESPONSABILIDADES:** aplicação do questionário de avaliação; processamento dos dados; divulgação dos resultados obtidos.

**ORIENTADORES / COORDENADORES DO ESTUDO**

**NOME:** NÚBIA VERCOSA FIGUEIREDO

**UNIDADE:** Serviço de anestesiologia do HUCFF-UFRJ

**CATEGORIA FUNCIONAL / REGIME DE TRABALHO:** médica anestesiologista; professora da Faculdade de Medicina da UFRJ.

**RESPONSABILIDADES:** elaboração do projeto de pesquisa; coordenação da pesquisa

**NOME:** Bruno Mendonça Barcellos, MSc.

**UNIDADE:** Serviço de anestesiologia do HUAP-UFF

**CATEGORIA FUNCIONAL / REGIME DE TRABALHO:** médico anestesiologista; 20 horas semanais.

**RESPONSABILIDADES:** processamento dos dados; análise estatística.

**NOME**: Ismar Lima Cavalcanti, MD,PhD.

**UNIDADE:** Serviço de anestesiologia do HUAP-UFF

**CATEGORIA FUNCIONAL / REGIME DE TRABALHO:** professor da Faculdade de Medicina da UFF

**RESPONSABILIDADES:** elaboração do projeto de pesquisa; coordenação da pesquisa.

### 2.6.2 DA INSTITUIÇÃO

Oferecer condições dignas e apropriadas para a execução das consultas ambulatoriais, internação, ato e recuperação anestésico-cirúrgico, bem como higiene pessoal, entretenimento, conforto e alimentação conforme regime próprio do Hospital Universitário Antônio Pedro (HUAP-UFF).

### 2.6.3 DO PATROCINADOR

O presente estudo não possui patrocinador.

## 2.7 DOS CRITÉRIOS PARA SUSPENDER OU ENCERRAR A PESQUISA

O ensaio será suspenso em qualquer ocasião caso surjam eventos adversos inesperados que justifiquem a suspensão do trabalho. Neste caso, será comunicado à Comissão de Ética local e ao CONEP.

## 2.8 LOCAL DA PESQUISA

A pesquisa ora proposta será desenvolvida no serviço de radiologia (2^o^. andar do Hospital Universitário Antônio Pedro - HUAP) da Universidade Federal Fluminense (UFF), localizado na Av. Marquês de Paraná, 303, Centro, Niterói/RJ.

## 2.9 INFRAESTRUTURA NECESSÁRIA AO DESENVOLVIMENTO DA PESQUISA

As biópsias prostáticas via transretal serão realizadas no serviço de radiologia do HUAP, após avaliação e seleção de pacientes ambulatoriais do Sistema Único de Saúde pelos médicos residentes em urologia, respeitando os critérios de inclusão e exclusão. As internações hospitalares ocorrerão na forma de hospital-dia (day clinic), com a recuperação e liberação do paciente ocorrendo no mesmo dia segundo avaliação anestésica.

Segundo normas do CFM (resolução n^o^. 1.802/96), os procedimentos ocorrerão em ambiente apropriado, em sala com saída de gases, aparelho de anestesia, monitorização continua e equipamentos de reanimação cardiopulmonar.

## 2.10 ORÇAMENTO FINANCEIRO

Toda a estrutura necessária ao desenvolvimento da pesquisa já existe no HUAP, uma vez que o procedimento já é realizado no setor. A pesquisa não onerará a instituição com gastos além dos previstos. Despesas com material de consumo (papel, impressão de fichas clínicas e de evolução) e exames laboratoriais especiais serão financiadas pelo responsável do ensaio, na forma de doação aos respectivos serviços. Demais custos eventuais serão arcados pelos pesquisadores principais.

## 2.11 PROPRIEDADE DAS INFORMAÇÕES GERADAS PELA PESQUISA

Detêm a propriedade intelectual das informações geradas pelo ensaio os pesquisadores responsáveis e os demais participantes do projeto.

## 2.12 PUBLICAÇÃO DOS RESULTADOS

Os resultados do projeto de pesquisa: “Mistura Óxido Nitroso-Oxigênio (50-50%) em Biópsia de Próstata Transretal Guiada por Ultrassonografia**”** serão tornados públicos, sejam eles favoráveis ou não.

## 2.13 USO E DESTINAÇÃO DO MATERIAL E/OU DADOS COLETADOS

Os dados coletados destinam-se exclusivamente aos fins especificados no presente projeto.

# 3 INFORMAÇÕES RELATIVAS AO SUJEITO DA PESQUISA

## 3.1 POPULAÇÃO A SER ESTUDADA

Serão selecionados 84 pacientes do gênero masculino, entre 18 e 75 anos, ASA I, II ou III, com suspeita de adenocarcinoma de próstata com indicação de biópsia prostática transretal em caráter eletivo.

## 3.2 CRITÉRIOS DE EXCLUSÃO DE INDIVÍDUOS

Serão excluídos do estudo pacientes em que houver:

- impossibilidade de relatar a intensidade da dor;
- incapacidade para inalar através do dispositivo;
- diagnóstico de hipertensão pulmonar;
- pneumopatia grave;
- cardiopatia NYHA 3 e 4.

## 3.3 PLANOS PARA RECRUTAMENTO E PROCEDIMENTOS

Os pacientes serão selecionados dentre aqueles que recorrem ao serviço de urologia do HUAP-UFF, por indicação médica ou vontade própria, obedecidos os critérios de inclusão e exclusão. Os pacientes serão instruídos quanto aos objetivos e procedimentos deste estudo, e após consentimento informado, avaliados clínica e laboratorialmente, de acordo com a rotina de exames pré-operatórios e estabelecimento do risco cirúrgico.

Abordagem dos pacientes já agendados, conforme a lista do Serviço de Readiologia do Hospital Universitário Antônio Pedro, no dia do exame por pesquisadores ou colaboradores.

## 3.4 TERMO DE CONSENTIMENTO DO PACIENTE

Os médicos residentes em anestesiologia, durante a seleção ambulatorial, irão explicar aos participantes do ensaio a natureza do estudo e responder todas as questões referentes ao mesmo. Antes de qualquer procedimento realizado com o paciente, o “Termo de Consentimento Livre e Esclarecido” será lido, assinado e datado pelo voluntário e pelo médico residente. Uma via deste Consentimento será fornecida ao paciente, e outra será mantida nos seus registros médicos. Uma anotação deverá também ser feita no “Formulário de Registro de Caso” (FRC) para confirmar que o Consentimento Livre e Esclarecido foi obtido antes de qualquer procedimento. Os elementos e o formato do Consentimento Livre e Esclarecido encontram-se no Anexo I.
