# Supplementary material for: A 50-50% mixture of nitrous oxide-oxygen in transrectal ultrasound-guided prostate biopsy: A randomized and prospective clinical trial
Source: PLoS One. 2018 Apr 27;13(4):e0195574. doi: 10.1371/journal.pone.0195574 (PMC5922537; doi:10.1371/journal.pone.0195574)
Supplement: S7 File — (DOCX) [file pone.0195574.s007.docx]

**FEDERAL FLUMINENSE UNIVERSITY**

**ANTÔNIO PEDRO UNIVERSITY HOSPITAL**

**ANESTHESIOLOGY SERVICE**

**RADIOLOGY SERVICE**

A 50-50% mixture of nitrous oxide-oxygen in transrectal ultrasound-guided prostate biopsy: a randomized and prospective clinical trial.

SEARCH PROTOCOL

(CNS n° 466/12)

**RESEARCHERS:**

  Md Gabriel da Silva Cazarim

**GUIDELINES:**

PhD Ismar Lima Cavalcanti

PhD Núbia Verçosa Figueiredo

**NITERÓI/RJ**

**2015**

SUMMARY

**1 INTRODUCTION………………………………………………………………… 4**

**2 DESCRIPTION OF THE RESEARCH………………………………………… 4**

**2.1 DESCRIPTION OF PURPOSES AND HYPOTHESES TO BE TESTED.. 4**

**2.2 SCIENTIFIC BACKGROUND AND DATA JUSTIFYING RESEARCH… 4**

**2.3 DETAILED AND ORDINATED DESCRIPTION OF THE RESEARCH PROJECT………………………………………………………………………….. 4**

**2.3.1 MATERIALS AND METHODS……………………………………………. 5**

**2.3.2 SELECTION, RANDOMIZATION AND BLINDNESS OF PATIENTS... 5**

**2.3.3 MEASURES OF MISSING………………………………………………… 5**

**2.3.4 CASE STUDY………………………………………………………………. 5**

**2.3.5 REGISTRATION AND DATA PRESENTATION……………………….. 6**

**2.3.6 STATISTICAL ANALYSIS………………………………………………... 6**

**2.3.7 EXPECTED RESULTS……………………………………………………. 6**

**2.3.8 REFERENCES……………………………………………………………....6**

**2.4 ANALYSIS OF RISKS AND BENEFITS…………………….…………... 7**

**2.5 TOTAL DURATION OF RESEARCH (FROM THE APPROVAL) …......7**

**2.6 RESPONSIBILITIES………………………………………………………. 8**

**2.6.1 RESEARCHER……………………………………………………..……... 8**

**2.6.2 INSTITUTION ……………………………………………………..………..9**

**2.6.3 SPONSOR ………………………………………………………………….. 9**

**2.7 CRITERIA FOR SUSPENDING OR FINDING RESEARCH…………….. 9**

**2.8 RESEARCH LOCATION…………………………………………………….. 9**

**2.9 INFRASTRUCTURE NEEDED FOR RESEARCH DEVELOPMENT….. 9**

**2.10 FINANCIAL BUDGET ……………………………………………………..10**

**2.11 PROPERTY OF INFORMATION GENERATED BY RESEARCH…… 10**

**2.12 PUBLICATION OF THE RESULTS ………………………………………10**

**2.13 USE AND DESTINATION OF MATERIAL AND / OR DATA COLLECTED ……………………………………………………………………………………….10**

**3 INFORMATION RELATED TO THE SUBJECT OF THE RESEARCH …..10**

**3.1 POPULATION TO BE STUDIED ……………………………………………11**

**3.2 EXCLUSION CRITERIA FOR INDIVIDUALS…………………………….. 11**

**3.3 PLANS FOR RECRUITMENT AND PROCEDURES……………………. 11**

**3.4 CONSENT TERM……………………………………………………………. 11**

# 1 INTRODUCTION

Transrectal ultrasound-guided prostate biopsy (TUSPB) is the standard method used for early diagnosis of cancer when associated with prostate-specific antigen (PSA) plasma levels.^1^ Although well tolerated by many patients, between 65 and 90% of men undergoing TUSPB complain of discomfort^2^ associated with pain. Several methods of analgesia and/or sedation have been proposed, including periprostatic^2-4^ or intraprostatic^5^ nerve block, topical anesthesia with lidocaine^6^ or EMLA^7^ at the puncture site, and general anesthesia with propofol and remifentanil.

The inhalation of 50-50% of nitrous oxide (N_2_O)-oxygen (O_2_) by the self-administration valve proposed in the present study is a good alternative to the routinely used methods in TUSPB since it is a safe, cost-effective technique that promotes analgesia on demand without the need of an anesthesiologist.^9.10^

.

# 2 RESEARCH DESCRIPTION

## 2.1 DESCRIPTION OF PURPOSES AND HYPOTHESES TO BE TESTED

The hypothesis of the present study is that the auto-inhalation of the mixture Nitrous Oxide-Oxygen (50-50%) decreases the pain intensity of the patient submitted to transretal prostate biopsy guided by ultrasonography.

Primary Objective:

- Assess the intensity of pain.

Secondary objectives:

- Assess the degree of satisfaction
- Measure the incidence of adverse events (nausea and vomiting, drowsiness, laughter, dizziness, euphoria or malaise)
- Evaluate hemodynamic changes.

The possibility of future standardization in HUAP is added.

## 2.2 SCIENTIFIC BACKGROUND AND DATA THAT JUSTIFY RESEARCH

**Nitrous oxide can be self-administered for analgesia in various procedures, such as intra-articular injection of drugs11, vascular access puncture12, sigmoidoscopy13, colonoscopy14, ophthalmologic procedures15 and prostate biopsy.16 Nitrous oxide has been used in emergencies, accident care and patient transport in ambulances.17**

## 2.3 DETAILED AND ORDINATED DESCRIPTION OF THE RESEARCH PROJECT

### 2.3.1 MATERIALS AND METHODS

The clinical trial will be performed at the Antônio Pedro University Hospital (HUAP), Niterói, RJ. At the present day, the procedure is performed in the outpatient clinics of the Hospital, without anesthetist.

An informed consent form will be signed by each of the volunteer participants, who were advised of the risks and benefits of the research. The patients will be divided into two groups according to a sequence of random numbers generated electronically through the program *GraphPad Prism®*. Forty-two patients will be allocated to the O_2_ group (C) and 42 to the N_2_O oxide group (NO). Group C will receive topical anesthesia in the anal canal (lidocaine hydrochloride jelly 2% - CRISTÁLIA Produtos Químicos e Farmacêuticos, Itapira, SP, Brazil) plus 100% oxygen inhalation under a facemask. In turn, the NO group will receive topical anal anesthesia (lidocaine hydrochloride jelly 2% gel - CRISTÁLIA Produtos Químicos e Farmacêuticos, Itapira, SP, Brazil) plus inhalation of the 50-50% N_2_O-O_2_ gas mixture (LIVOPAN®, Linde Gases, Rio de Janeiro, RJ, Brazil) through a self-administration valve.

An anesthesiologist will follow the examinations in compliance with the resolution of the Federal Council of Medicine (CFM) No. 1.802 / 2006, without being able to intervene in the analgesia proposed by the randomization. A visual analogue scale (EVA) of 0-10 pain and 0-10 satisfaction will be presented to the patients before the procedure and answered after the procedure. The group receiving the N2O-O2 mixture will be evaluated for the incidence of nausea, vomiting, dizziness, hemodynamic changes, laughter crisis, drowsiness during the examination.

### 2.3.2 SELECTION, RANDOMIZATION, AND BLINDNESS OF PATIENTS

All patients who are assigned to the BPTU at HUAP during the period of the survey will be selected, with the exception of those who fit the exclusion criteria. Patients will be randomly assigned to one of two anesthetic technique groups, according to randomly generated sequence of random numbers. Because it is a double blind study, patients and the researcher performing the questionnaire will not know which randomized group.

### 2.3.3 ENDPOINT MEASURES

Primary endpoint: decrease in pain frequency and intensity during BPTU.

Secondary outcome: determination of the incidence of adverse effects and hemodynamic changes, besides the knowledge of the degree of satisfaction of the patients with the analgesia performed..

### 2.3.4 CASUISTRY

A 30% difference (from 80 to 50%) in the pain score classified as intense (EVA> 7) reported by the patients after the BPTU was performed as a primary endpoint, a level of statistical significance of 5% (α = 0.05) and a test power of 80% (error β = 0.20), 2 groups of 38 patients became necessary. Predicting censoring of the data obtained, we increased the sample by 10%, resulting in the formation of 2 equally sized groups with 42 patients each (n = 84). A value of p <0.05 will be considered statistically significant.

### 2.3.5 REGISTRATION AND DATA PRESENTATION

The data generated by the research will be registered and presented in its own document (Annexes II, III and IV), in the possession of all research teams, except for the "Blind and Randomization Form" (Annex IV), which will be exclusively owned by the pilot researcher. These data will serve as a basis for statistical analysis of the entire work.

### 2.3.6 STATISTICAL ANALYSIS

At the end of the experimental and data collection phase, all information generated by the test will be submitted to statistical analysis in SPSS v.19.0 (IBM, New York, USA). Values will be expressed as means, medians or number of patients. The normal distribution pattern of the data will be tested using the Shapiro-Wilk method. Parametric data will be analyzed using the Student's t-test to compare the significance of means between groups. Non-parametric data will be compared using the Wilcoxon test between groups. A p value <0.05 will be considered statistically significant.

### 2.3.7 EXPECTED RESULTS

The purpose of the clinical trial is to reduce the intensity of pain and increase the comfort and satisfaction index during the BPTU, in the period of low rate of adverse events.

### 2.3.8 REFERENCES

1. Uno H, Nakano M, Ehara H, Degushi T. Indications for 14-core transrectal ultrasound-guided prostate biopsy. Urology. 2008;71(1):23-27.
2. Inal G, Yazici S, Adnan O, Ozturk B, Kosan M, Cetinkaya M. Effect of periprostatic nerve blockade before transrectal ultrasound-guided prostate biopsy on patient comfort: A randomized placebo controlled study. Int J Urol. 2004;11:148-151.
3. Ozveri H, Cevik I, Dillioglugil O, Akdas A. Transrectal periprostatic lidocaine injection anesthesia for transrectal prostate biopsy: a prospective study. Prostate Cancer and Prostatic Dis. 2003;6(4):311-314.
4. Autorino R, de Sio M, di Lorenzo G, Damiano R, Perdona S, Cindolo L, D'Armiento M. How to decrease pain during transrectal ultrasound guided prostate biopsy: a look at the literature. J Urol. 2005;174(6):2091-2097.
5. Bingqian L, Peihuan L, Yudong W, Jinxing W, Zhiyong W. Intraprostatic local anesthesia with periprostatic nerve block for transrectal ultrasound guided prostate biopsy. J Urol 2009; 182(2):479-483.
6. Kubo Y, Kawakami S, Numan N, Takazawa R, Fujii Y, Masuda H, Tsujii T, Kihara K. Simple and effective local anesthesia for transperineal extended prostate biopsy: Application to three-dimensional 26-core biopsy. Int J Urol. 2009; 16: 420-423.
7. Basar H, Basar M, Ozan S, Akpinar S, Basar H, Batislam E. Local anesthesia in transrectal ultrasound-guided prostate biopsy: EMLA cream as a new alternative technique. Scand J Urol and Nephrol. 2005; 39: 130-134.
8. Kang SG, Tae BS, Min SH, Ko YH, Kang SH, Lee JG, Kim JJ, Cheon J. Efficacy and cost analysis of transrectal ultrasound-guided prostate biopsy under monitored anesthesia. Asian J Androl. 2011; 13(5): 724-727.
9. Pita CP, Pazmiño S, Vallejo M, Salazar-Pousada DS, Hidalgo L, Pérez-López FR, Chedraui P; Research Group for the Birth Humanization Project of Enrique C. Sotomayor Hospital. Inhaled intrapartum analgesia using a 50–50 % mixture of nitrous oxide–oxygen in a low-income hospital setting. Arch Gynecol Obstet. 2012; 283(3):627-631.
10. Emmanouil, DE; Quock, RM. Advances in understanding the actions of nitrous oxide. Anesth Prog. 2007; 54(1): 9–18.
11. Uziel Y, Chapnick G, Rothsxhild M, Tauber T, Press J, Harel L, Hashkes PJ. Nitrous oxide sedation for intra-articular injection in juvenile idiopathic arthritis. Pediatr Rheumatol Online J. 2008; 15(6):1-4.
12. Gerhardt RT, King KM, Wiegert RS. Inhaled nitrous oxide versus placebo as an analgesic and anxiolytic adjunct to peripheral intravenous cannulation. Am J Emerg Med. 2001; 19(6): 492–494.
13. Harding TA, Gibson JA. The use of inhaled nitrous oxide for flexible sigmoidoscopy: a placebo-controlled trial. Endoscopy*.* 2000; 32(6): 457–460.
14. Forbes GM, Collins BJ*.* Nitrous oxide for colonoscopy: a randomized controlled study. Gastrointestinal Endoscopy*.* 2000;51(3): 271–277.
15. Cook HL, Newsom RS, Mensah E, Saeed M, James D, Ffytche TJ. Entonox as an analgesic agent during panretinalphotocoagulation. Br J Ophtalmol. 2002; 86(10): 1107–1108.
16. Masood J, Shah N, Lanes T, Andrews H, Simpson P, Barua JM. Nitrous oxide (entonox) inhalation and tolerance of transrectal ultrasound guided prostate biopsy: a double-blind randomized controlled study. J Urol. 2002, 168(1):116–120.
17. Baskett PJ. Use of Entonox in the ambulance service. Br Med J. 1970; 4(2): 41–43.

## 2.4 ANALYSIS OF RISKS AND BENEFITS

The use of the nitrous oxide-oxygen mixture (50-50%) in anesthesia offers few side effects to the patients, being the most known: sedation, dizziness. nausea, and euphoria. In contrast, it offers good quality analgesics, presenting itself as good choice for outpatient procedures.

In addition, patients in group C and ON group are exposed to the risks of the procedure itself: hemospermia, hematuria, rectal bleeding, urinary tract infection and prostatitis.

## 2.5 TOTAL DURATION OF RESEARCH (FROM THE APPROVAL)

The data collection phase (BPTU) will start in February / 2015 and forecast of 4 (four) tests per week due to high demand, the duration of the survey should be 12 (twelve) months and may extend for a further 6 (six months. The entire data collection phase should end at the end of the first half of 2015, leaving only the processing of the samples and statistical analysis.

## 2.6 RESPONSIBILITIES

### 2.6.1 RESEARCHER

**MAIN RESEARCHER**

**NAME**: Gabriel da Silva Cazarim, MD.

**DEPARTMENT:** HUAP-UFF anesthesiology service

**CATEGORIA FUNCIONAL:** resident physician / specializing in anesthesiology; 60 hours / week.

**RESPONSABILITIES:** selection and randomization of the sample; administration of the tested gas

**GUIDELINES**

**NAME:** NÚBIA VERCOSA FIGUEIREDO

**DEPARTMENT:** HUCFF-UFRJ anesthesiology service

**CATEGORIA FUNCIONAL:** medical anesthesiologist; professor at the Medical School of UFRJ.

**RESPONSABILITIES:** elaboration of the research project; research coordination

**NAME**: Ismar Lima Cavalcanti, MD,PhD.

**DEPARTMENT:** HUAP-UFF anesthesiology service

**CATEGORIA FUNCIONAL:** professor at the Medical School of UFF

**RESPONSABILITIES:** elaboration of the research project; research coordination

### 2.6.2 INSTITUTION

To offer dignified and appropriate conditions for the execution of outpatient consultations, hospitalization, act and anesthetic-surgical recovery, as well as personal hygiene, entertainment, comfort and feeding according to the own regime of Hospital Universitário Antônio Pedro (HUAP-UFF).

### 2.6.3 SPONSOR

This study has no sponsor.

## 2.7 CRITERIA FOR SUSPENDING OR ENDING THE RESEARCH

## The trial will be suspended at any time in the event of unexpected adverse events that justify the suspension of work. In this case, it will be communicated to the local Ethics Committee and CONEP

## .2.8 LOCATION OF THE RESEARCH

The research proposed here will be developed at the Radiology Department (2nd floor of the Antônio Pedro University Hospital - HUAP), Federal University of Fluminense (UFF), located at Av. Marquês de Paraná, 303, Centro, Niterói / RJ.

## 2.9 INFRASTRUCTURE NEEDED FOR RESEARCH DEVELOPMENT

The transrectal prostate biopsies will be performed in the radiology department of the HUAP, after evaluation and selection of outpatients of the Unified Health System by doctors residing in urology, respecting the inclusion and exclusion criteria. Hospital admissions will take place as a day-clinic, with recovery and release of the patient occurring on the same day according to anesthesia evaluation.

According to CFM regulations (Resolution No. 1.802 / 96), procedures will be performed in an appropriate environment, in an exhaust room, anesthesia machine, continuous monitoring and cardiopulmonary resuscitation equipment.

## 2.10 FINANCIAL BUDGET

All the structure necessary for the development of the research already exists in HUAP, once the procedure is already carried out in the sector. The research will not burden the institution with expenses beyond those anticipated. Expenditure on consumables (paper, printing of clinical and evolution records) and special laboratory tests will be financed by the test leader, in the form of a donation to the respective services. Other eventual costs will be borne by the principal researchers.

## 2.11 PROPERTY OF THE INFORMATION GENERATED BY THE RESEARCH

The intellectual property of the information generated by the trial holds the responsible researchers and the other participants in the project.

## 2.12 PUBLICATION OF RESULTS

The results of the research project " A 50-50% mixture of nitrous oxide-oxygen in transrectal ultrasound-guided prostate biopsy: a randomized and prospective clinical trial." will be made public, whether they are favorable or not.

## 2.13 USE AND DESTINATION OF MATERIAL AND / OR DATA COLLECTED

The data collected is exclusively for the purposes specified in this project.

# 3 INFORMATION CONCERNING THE SUBJECT OF THE RESEARCH

## 3.1 POPULATION TO BE STUDIED

A total of 84 male patients, aged between 18 and 75 years, ASA I, II or III, with suspected adenocarcinoma of the prostate with indication of elective transrectal prostatic biopsy, will be selected.

## 3.2 INDIVIDUAL EXCLUSION CRITERIA

Patients will be excluded from the study if there are:

- unable to report the intensity of pain;

- Inability to inhale through the device;

- diagnosis of pulmonary hypertension;

- severe pneumopathy;

- NYHA heart disease 3 and 4.

## 3.3 PLANS FOR RECRUITMENT AND PROCEDURES

Patients will be selected from among those who use the urology service of HUAP-UFF, by medical indication or own will, obeying the inclusion and exclusion criteria. Patients will be instructed about the objectives and procedures of this study, and after informed consent, evaluated clinically and laboratorially, according to the routine of preoperative exams and establishment of surgical risk.Abordagem dos pacientes já agendados, conforme a lista do Serviço de Readiologia do Hospital Universitário Antônio Pedro, no dia do exame por pesquisadores ou colaboradores.

## 3.4 CONSENT TERM

Physicians residing in anesthesiology, during outpatient screening, will explain to the trial participants the nature of the study and answer any questions regarding it. Before any procedure performed with the patient, the "Informed Consent Form" will be read, signed and dated by the volunteer and the resident physician. One way of this Consent will be provided to the patient, and another one will be kept in your medical records. An annotation should also be made on the "Case Registration Form" (FRC) to confirm that the Free and Informed Consent was obtained prior to any procedure.
